# Promoting a healthy diet and physical activity in adults with intellectual disabilities living in community residences: Design and evaluation of a cluster-randomized intervention

**DOI:** 10.1186/1471-2458-10-761

**Published:** 2010-12-13

**Authors:** Liselotte Schäfer Elinder, Helena Bergström, Jan Hagberg, Ulla Wihlman, Maria Hagströmer

**Affiliations:** 1 Division of Intervention and Implementation Research, Department of Public Health Sciences, Karolinska Institutet, Stockholm, Sweden; 2 Division of Physiotherapy, Department of Neurobiology, Care Sciences and Society, Karolinska Institutet, Stockholm, Sweden

## Abstract

**Background:**

Many adults with intellectual disabilities have poor dietary habits, low physical activity and weight disturbances. This study protocol describes the design and evaluation of a health intervention aiming to improve diet and physical activity in this target group. In Sweden, adults with intellectual disabilities often live in community residences where the staff has insufficient education regarding the special health needs of residents. No published lifestyle interventions have simultaneously targeted both residents and staff.

**Methods/Design:**

The intervention is designed to suit the ordinary work routines of community residences. It is based on social cognitive theory and takes 12-15 months to complete. The intervention includes three components: 1) Ten health education sessions for residents in their homes; 2) the appointment of a health ambassador among the staff in each residence and formation of a network; and 3) a study circle for staff in each residence. The intervention is implemented by consultation with managers, training of health educators, and coaching of health ambassadors. Fidelity is assessed based on the participation of residents and staff in the intervention activities. The study design is a cluster-randomised trial with physical activity as primary outcome objectively assessed by pedometry. Secondary outcomes are dietary quality assessed by digital photography, measured weight, height and waist circumference, and quality of life assessed by a quality of life scale. Intermediate outcomes are changes in work routines in the residences assessed by a questionnaire to managers. Adults with mild to moderate intellectual disabilities living in community residences in Stockholm County are eligible for inclusion. Multilevel analysis is used to evaluate effects on primary and secondary outcomes. The impact of the intervention on work routines in community residences is analysed by ordinal regression analysis. Barriers and facilitators of implementation are identified in an explorative qualitative study through observations and semi-structured interviews.

**Discussion:**

Despite several challenges it is our hope that the results from this intervention will lead to new and improved health promotion programs to the benefit of the target group.

**Trial registration number:**

ISRCTN33749876

## Background

Dietary habits, physical activity and obesity are strong modifiable risk factors for chronic diseases such as cardiovascular diseases, diabetes and some cancers [1]. People with intellectual disabilities (ID) often have poor dietary habits [2,3], low physical activity [4,5], and weight disturbances [4-8]. According to a report from the Swedish National Institute of Public Health people with ID carry a higher disease burden than the population in general and interventions directed at these risk factors could be an important way to improve health in this group [9].

In Sweden, individuals with ID who live in community residences are entitled to assistance in everyday life, but the staff often has insufficient education regarding the special needs of people with disabilities [10]. An ID involves a reduced short term memory and a reduced ability for abstraction which increases the risk of making unhealthy choices in an obesogenic environment [11]. IDs are usually categorised as mild, moderate, severe or profound [12]. The categories are arbitrary divisions of a complex continuum, and cannot be defined with absolute precision.

Few lifestyle interventions targeting people with ID have been published. Health programmes, including education, exercise, and in a few cases also stress reduction, have shown modest decreases in BMI [13-17]. In two of those studies, significant improvements were seen in the quality of life in the intervention group after completing the programme [13,16]. In another intervention, where staff received training in meal preparation and weekly supervisor feedback, routines improved and were maintained for up to one year [18]. Positive health changes, in terms of weight loss and decreased blood pressure, were seen in the residents. To our knowledge no interventions have been published targeting both staff and residents in the same community residence. In order to promote sustainability of the intervention it is designed to suit into the normal work routines of community residences. The aim of this paper is to describe and explain the design and evaluation of this health intervention targeting people with ID. The description of the study protocol follows the checklist of the CONSORT statement for cluster-randomized trials [19].

## Methods and design

### Study objectives

1) To study effects of a health intervention on residents' diet quality, physical activity, body mass index (BMI) and quality of life.

2) To study improvements at residence level in work routines and opportunities for healthy diets and physical activity.

3) To describe and analyse barriers and facilitators in the implementation of the intervention.

### Hypothesis

We hypothesise that an educational approach directed both at the residents and staff will strengthen the knowledge and skills of the residents to improve their diet and increase physical activity, as well as the ability of staff to provide a supportive social and physical environment for making healthy choices.

### Setting and target group

Adult men and women with mild to moderate ID who live in community residences in Stockholm County are eligible for inclusion. There are approximately 500 such residences. For a residence to be included, at least three subjects in each residence have to agree to participate. All participants need to have the ability to understand simple information about the study and to decide whether they want to participate or not. A letter of invitation is sent to managers, who are asked to contact their subordinated community residences in Stockholm County. After notification of interest from residences, residents receive an easy-to-read letter with information about the purpose of the study and about the intervention itself. Participants who express interest to participate are informed verbally and in writing, and written consent is obtained. Staff at the residences as well as trustees or legal guardians also receive written and verbal information about the intervention and the purpose of the study. All participants are rewarded with a cinema ticket. Quantitative data will be presented in aggregated form. Qualitative data will be abstracted into themes and illustrated by quotations from the interviews and observations. The data will be treated as strictly confidential and it will not be possible to identify individuals or residences. Ethical permission for this study has been obtained from the Regional Ethical Review Board in Stockholm County No. 2009/1332-31/5.

### Planning of the intervention and development of materials

When planning this intervention we employed the step-by-step approach as described by Fraser et al. [20]. In step 1 we have specified the problem and defined our problem theory. Based on previous research and practitioner's experience, we have identified poor dietary habits, low physical activity and weight disturbances among adults with ID. In addition, the low educational level of staff working in community residences in general has been identified as a barrier to healthy lifestyles among the target group. Our goal with the intervention is therefore to improve diet and physical activity of the residents as well as knowledge and skills of both residents and the staff. The intervention is based on social cognitive theory (SCT). This theory explains behaviour in terms of a triadic, dynamic, and reciprocal model in which behaviour, personal factors, and environmental influences all interact [21]. According to this theory, we aim to improve health behaviours through both personal factors (knowledge, skills, preferences, self-efficacy) of residents as well as through improvements in their social and physical environment, which is very much dependent on knowledge, skills and work routines of the staff.

In step 2 of intervention planning, core components of the intervention are identified and programme materials are developed and pretested. "Fokus hälsa" (Focus health) is a newly developed material with ten themes for use by staff. It aims to increase the knowledge and skills of residence staff with regard to diet, physical activity and health and is based on the principles of peer education [22]. The themes were developed in discussions with managers of community residences and on the basis of their knowledge of the needs of the target group. The themes are: 1) Health and quality of life; 2) Autonomy and ethics; 3) National recommendations concerning diet and health and information in society; 4) Healthy dietary habits; 5) Physical activity for health; 6) Availability and accessibility; 7) Habits and attitudes; 8) Motivation and support for behavioral change; 9) Cooperation; and 10) How to sustain good work. Each theme includes an introductory text, which is read before the meeting, and at the end there are three suggested exercises: 1) Questions for discussion; 2) Identification of strengths and weaknesses in work routines; and 3) Making agreements about new and improved work routines.

A health education material "Hälsokörkortet" (Driver's licence for health) has been developed specifically for people with ID and pretested and revised by "Studieförbundet Vuxenskolan", a national educational association for adults. The material includes ten educational sessions within five areas: diet, physical activity, culture/aesthetics, mental health and stress relief.

### Intervention components

The intervention takes 12-15 months to complete and includes three main components: 1) Ten health education sessions for residents; 2) the appointment of a health ambassador among the staff in each residence, including four network meetings; and 3) a study circle for staff based on the principles of peer education.

#### Health education for residents

The aim of the health education is to support residents to make lifestyle changes in an easy and positive way in their everyday life, by increasing their knowledge, preferences, skills and self-efficacy. Participants get the possibility to try new foods and activities, and are assigned home work. Ten sessions are carried out in the residences by a health educator from "Studieförbundet Vuxenskolan" using the educational material "Hälsokörkortet" (Driver's licence for health). The material includes themes and detailed instructions for the educator for each of the ten sessions. Each session is supposed to last for 2 × 45 minutes and comprises of a discussion, a theme activity, testing of healthy food, physical activity and homework.

#### Health ambassadors

Ambassadors for various issues are common among the staff in community residences and we wanted to build on this practice. Therefore, in every participating community residence a health ambassador is appointed among staff members. The task of the health ambassador is to provide health information to colleagues and inspire them and to plan and organise health promoting activities for residents. The health ambassador receives coaching by the research team on issues regarding diet, physical activity and health and gets the possibility to exchange knowledge and experiences during network meetings with health ambassadors from the other residences. Network meetings are arranged four times during the intervention. The first meeting is an introductory meeting and following meetings focus on themes chosen by the ambassadors.

#### Study circle for staff

The aim of the study circle is to increase the knowledge and skills of staff in the area of health promotion in order to empower them to improve work routines and the social and physical environment of residents. Normally, staff in community residences meet once every second or fourth week. We decided that these meetings would be a good opportunity for the staff to conduct this study circle using the study material "Fokus Hälsa" (Focus Health). All the staff in every community residence, including the health ambassador, comes together to discuss health issues, based on the principles of peer education [22]. A discussion leader is appointed and the group discusses and does the exercises according to the instructions in the material.

### Implementation components

**Figure 1:**
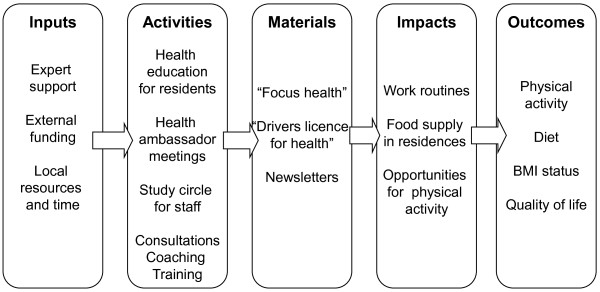
**Logic model of the intervention in community residences**.

Implementation components or drivers are factors which enable practitioners to implement the intervention as intended [23]. Typical components used in this programme are consultations with managers, training and coaching of staff. First, managers and staff in the residences are invited to an introductory meeting discussing the entire intervention with the research team. Second, the health educators are trained by the research staff together with experienced educators from "Studieförbundet Vuxenskolan" for one day regarding how to use the material "Hälsokörkortet" (Drivers licence for health). During the entire intervention period the health ambassadors receive coaching on demand from the research group via personal visits, mail, telephone, and at network meetings. Newsletters are sent to all residences to keep staff and residents up to date with the overall state of the project and important news. Figure 1 shows the logic model of the intervention explaining the hypothetical causal chain that may lead to behaviour changes of the target group.

### Evaluation design

Step 3 in intervention research [20] constitutes the evaluation of the intervention with regard to individual outcomes and setting level impacts. Because we are targeting both residents and staff the study is designed as a cluster-randomised trial. After baseline measurements are completed, residences are randomised to an intervention group and to a waiting list-control group by a computer-generated list of random numbers done by the statistician, and concealed from the research staff, which enrols the participants. Evaluation is done by mixed methods, using both quantitative and qualitative methods.

#### Intervention outcomes

All outcomes at individual level are assessed at baseline, directly after the end of the intervention and again after 6 months. The primary intervention outcome is physical activity, which is assessed by pedometry (Yamax 200). The Yamax 200 pedometer has shown high agreement with accelerometer-measured physical activity [24,25] and is recommended for research purposes [26]. A previous study on adults with ID has shown that three days of measurement is needed to predict the usual weekly number of steps per day [27]. The participants receive the pedometer and, together with staff in residences, get practical training as well as verbal and easy to read instructions from the research staff on how to wear it (attached to a belt or lining around the waist, vertically in line with the knee), how to record the number of steps per day and how to reset the pedometer for the next day of measurement. The participants are asked to wear the pedometer for seven consecutive days, but for the analysis we will include participants that have at least three days of measurements. The outcome measure is average total steps per day as an indicator of total physical activity.

Dietary quality, which is a secondary outcome, is assessed by personal digital photography, a method which has been developed and validated for this project and will be published. Each participant, as well as residence staff, is instructed on how to use the camera (Canon PowerShot A480) and receives practical training in taking pictures. Photos are taken by the participants themselves of all foods and beverages consumed during three days and staff is encouraged to remind participants to take photos or to assist, if necessary. Outcome measures are: 1) Intake occasions of indicator foods and beverages (fruit and berries, vegetables, low nutrient density foods and beverages, and beverages excluding water); 2) Meal quality assessed in comparison to food-based dietary guidelines visualised as the plate model [28] and; 3) Dietary diversity covering nine core food groups.

It is important to assess the effects of health promotion efforts on the quality of life, which may improve or worsen as a consequence of the intervention. Quality of life, a secondary outcome, among people with ID is assessed by a multi-factorial quality of life scale which has been developed within this project, because no suitable scale could be identified from the literature. We selected questions from various quality of life questionnaires and reconstructed and pretested them. The questions include information about quality of life within six domains (home, food and meals, leisure, family and friends, physical health and mental health) and have three response alternatives; good, average or bad. The questions in the scale are read to participants in the form of a structured interview by the research team. Residence staff is asked not to be present during the interview, only if they think it is necessary for the sake of the respondent. The scale will be tested for its psychometric properties in participants in the study according to Kline el al. [29].

Height, weight and waist circumference are secondary outcomes and are measured by the research staff, at the residence. Height is measured to the nearest cm in a standardized way using SECA stadiometer (214). Weight is measured using a digital scale (SECA Robusta 813) to the nearest 0.5 kg with light clothing, and the body mass index (BMI kg/m^2^) is calculated. Waist circumference is measured to the nearest cm at the midpoint between the lowest rib and the iliac crest at the end of expiration. Participants answer questions regarding age, country of birth, occupation, family and physical functional limitation. If necessary, the staff gives supplementary information.

#### Impacts at setting level

Changes in health promotion work routines and healthy living opportunities in community residences are monitored by self-assessment by residence managers. We developed a questionnaire called Work routines for meals, physical activity and health (Additional file 1). It includes 26 items structured into 3 sections: General health promotion, food and meals, and physical activity. The questionnaire has undergone cognitive response testing but is not otherwise validated. The questionnaire is answered at baseline, after completed intervention and again at 6 months follows up in both intervention and control residences.

#### Fidelity criteria

Fidelity is defined by the extent to which a programme is implemented as intended, and the effectiveness of an intervention is related to fidelity [30]. Fidelity is assessed based on participation of residents and staff in the intervention activities. Fidelity is assessed by counting the number of times that residents and staff participate in health education and the study circle, respectively. Residents' participation is documented by the education leaders, staff participation is self-monitored and attendance at health ambassador's network meetings is documented by the research staff. A scoring system is developed with a 3-grade score, high, middle and low participation. Additional fidelity assessment is performed for the study circle for residence staff. A score is given to each residence based on the number of themes they have covered and to which extent they have made agreements about new and improved work routines.

#### Statistical power

Calculation of power is based on the assumption of an average 25% increase in physical activity, assessed as steps per day by pedometry. No Swedish data were available so we used data from Peterson et al [31] from the USA for adults with mild to moderate intellectual disabilities, who on average achieved 6621 ± 3366 steps per day. Calculations were performed with the "Sample size calculator for cluster randomized trials" [32]. The calculation was 2-sided, and power was set to 80%, the significance level to 5%, and cluster size to five individuals. The calculation shows that 32 community residences are needed to detect a significant change in physical activity of 25% between the intervention and control group.

#### Data analysis

Data analysis will be performed by the statistician who is blinded to the intervention group assignment. At individual level, parametric and non-parametric tests are used to compare groups at baseline, depending on the distributions of the quantitative variables. In order to account for clustering of the data, multilevel analysis is used to evaluate the effects of the intervention on relevant outcomes. Two levels are defined in the analysis: 1) individual and 2) residence. Linear and logistic regression models are used to study the effect on the outcome variables physical activity, intake occasions of indicator foods, meal quality, dietary diversity, body weight status, BMI and quality of life. Intervention outcome will be evaluated in relation to the intervention dose (fidelity score) but also to the intention to treat principle.

To evaluate the impact of the intervention on work routines and opportunities for a healthy diet and physical activity in community residences ordinal regression will be used for the data derived from the questionnaire Work routines for meals, physical activity and health (Additional file 1). Multinomial logistic regression might also be used if it is the categories and not the order of the categories per se that is of importance for the results. If the dependent variable can be adequately dichotomised, we will use Poisson regression with robust error variance (modified Poisson regression) to estimate risk ratios (relative risks).

#### Evaluation of barriers and facilitators of implementation

To define and analyse barriers and facilitators related to the process of implementation an explorative qualitative study will be performed. Interviews are often used when the aim is to gain a deep understanding of a phenomenon where not much is previously known [33,34], whereas observations are suitable to use when the aim is to explore what actually happens during the session [35]. Although it is possible to successfully conduct interviews with people with ID it involves several difficulties. A low level of responsiveness to open-ended questions is one [36], which is why observation is chosen as method for the health education sessions for residents. During or directly after the observations extensive notes will be taken by the observer.

Semi-structured interviews [37] are conducted with health ambassadors and managers after completed intervention. The number of informants depends on when saturation of information is attained e.g. when no further information is added which is usually after about 15-20 interviews [37]. The interviews will be recorded and transcribed verbatim. For analyses of the interviews and observations a thematic analysis will be used as described by Malterud [38]. Thematic analysis is suitable for explorative analysis in order to develop concepts or themes that are unknown prior to the analysis. The analysis procedure is an iterative process between formulated themes and original data. Trustworthiness of the study will be assured by a thorough description of the methods used, as well as inter-subjective agreement between different researchers in defining the different themes [39]. Anonymous quotations from the original interviews and observations will be used to illustrate the different themes in order to further enhance the credibility.

## Discussion

To our knowledge this is the first intervention study addressing diet and physical activity habits in people with ID which is simultaneously targeting staff and residents. People with different kinds of disabilities constitute a vulnerable group with a large and avoidable chronic disease burden and therefore health interventions are badly needed [9]. Nevertheless, there are several challenges in working with this target group. First, in our experience it is not so easy to recruit residences because they are not only homes but also workplaces for the staff and managers, who are busy doing their job. In addition, not all residents are willing to participate in a study, due to difficulties in understanding the content of the intervention and the consequences of participation. Second, methods for assessment of outcomes as well as programme materials for the intervention have to be adapted to the limited cognitive abilities of the target group. A new method for assessment of diet quality, diet diversity and intake occasions of indicator foods by digital photography has been validated within this project and will be published. Conducting interviews and using questionnaires among people with ID in order to assess quality of life is difficult due to cognitive limitations, and no suitable scale was found in the literature. A scale was developed by using relevant questions from different quality of life questionnaires. The psychometric properties of this scale will be assessed within the frame of this intervention.

In general it is hard to achieve weight loss in healthy adults through interventions targeting diet and physical activity [40]. We therefore do not expect to see significant weight losses in the intervention group. In addition, the study is not powered for this purpose. However, we hope to see changes in work routines at residence level as well as opportunities for healthy eating and physical activity for residents. We also expect improvements in residents' health behaviours.

There are also a number of ethical challenges in this intervention because people with ID are in need of professional care, but also have the basic right to autonomy and self-determination [41]. This will be dealt with in a separate qualitative study within this project. It is our hope that the results from this intervention will lead to new and improved health promotion programs to the benefit of the target group. Final results from the intervention study are expected in 2013.

## Competing interests

The authors declare that they have no competing interests.

## Authors' contributions

LSE and HB conceived the study, applied for funding, designed the intervention and the evaluation and are responsible for implementation and data collection. LSE prepared the initial draft of the manuscript. UW is responsible for the qualitative methods and the analysis. JH is responsible for the statistical analysis. MH is responsible for assessment and analysis of physical activity. All authors critically reviewed the manuscript and approved the final draft.

## Pre-publication history

The pre-publication history for this paper can be accessed here:

http://www.biomedcentral.com/1471-2458/10/761/prepub

## Supplementary Material

Additional file 1 **Work routines for meals, physical activity and health**. A questionnaire for administrators and managers of community residences concerning health promotion work routines Click here for file

## References

[B1] Branca F, Nikogosian H, Lobstein T Impact of obesity on health The challenge of obesity in the WHO European Region and the strategies for response 2007 Copenhagen: World Health Organization 20 27

[B2] Adolfsson P Sydner YM Fjellstrom C Lewin B Andersson A Observed dietary intake in adults with intellectual disability living in the community Food Nutr Res 2008 52 19109653 10.3402/fnr.v52i0.1857 PMC2596732

[B3] Draheim CC Stanish HI Williams DP McCubbin JA Dietary intake of adults with mental retardation who reside in community settings Am J Ment Retard 2007 112 5 392 400 10.1352/0895-8017(2007)112[0392:DIOAWM]2.0.CO;2 17676962

[B4] Emerson E Underweight, obesity and exercise among adults with intellectual disabilities in supported accommodation in Northern England J Intellect Disabil Res 2005 49 Pt 2 134 143 10.1111/j.1365-2788.2004.00617.x 15634322

[B5] Robertson J Emerson E Gregory N Hatto C Turner S Kessissoglou S Hallam A Lifestyle related risk factors for poor health in residential settings for people with intellectual disabilities Res Dev Disabil 2000 21 6 469 486 10.1016/S0891-4222(00)00053-6 11153830

[B6] Bhaumik S Watson JM Thorp CF Tyrer F McGrother CW Body mass index in adults with intellectual disability: distribution, associations and service implications: a population-based prevalence study J Intellect Disabil Res 2008 52 Pt 4 287 298 10.1111/j.1365-2788.2007.01018.x 18339091

[B7] Moran R Drane W McDermott S Dasari S Scurry JB Platt T Obesity among people with and without mental retardation across adulthood Obes Res 2005 13 2 342 349 10.1038/oby.2005.46 15800293

[B8] Hove O Weight survey on adult persons with mental retardation living in the community Res Dev Disabil 2004 25 1 9 17 10.1016/j.ridd.2003.04.004 14733973

[B9] Arnhoff Y Onödig ohälsa. Hälsoläget för personer med funktionsnedsättning. [Unnecessary poor health. Health status for people with disabilities.] 2008 Östersund: Swedish National Institute of Public Health

[B10] Lägesrapport 2003. [Disability Care. Status report 2003] 2003 Stockholm: The National Board of Health and Welfare, Handikappomsorg

[B11] Elinder LS Jansson M Obesogenic environments--aspects on measurement and indicators Public Health Nutr 2009 12 3 307 315 18498677 10.1017/S1368980008002450

[B12] International Statistical Classification of Diseases and Related Health Problems 2007 10 Geneva: WHO

[B13] Bazzano AT Zeldin AS Diab IR Garro NM Allevato NA Lehrer D The Healthy Lifestyle Change Program: a pilot of a community-based health promotion intervention for adults with developmental disabilities Am J Prev Med 2009 37 6 Suppl 1 S201 208 10.1016/j.amepre.2009.08.005 19896020

[B14] Chapman MJ Craven MJ Chadwick DD Following up fighting fit: the long-term impact of health practitioner input on obesity and BMI amongst adults with intellectual disabilities J Intellect Disabil 2008 12 4 309 323 10.1177/1744629508100557 19074936

[B15] Ewing G McDermott S Thomas-Koger M Whitner W Pierce K Evaluation of a cardiovascular health program for participants with mental retardation and normal learners Health Educ Behav 2004 31 1 77 87 10.1177/1090198103259162 14768659

[B16] Heller T Hsieh K Rimmer JH Attitudinal and psychosocial outcomes of a fitness and health education program on adults with down syndrome Am J Ment Retard 2004 109 2 175 185 10.1352/0895-8017(2004)109<175:AAPOOA>2.0.CO;2 15000672

[B17] Marshall D McConkey R Moore G Obesity in people with intellectual disabilities: the impact of nurse-led health screenings and health promotion activities J Adv Nurs 2003 41 2 147 153 10.1046/j.1365-2648.2003.02522.x 12519273

[B18] Kneringer MJ Page TJ Improving staff nutritional practices in community-based group homes: evaluation, training, and management J Appl Behav Anal 1999 32 2 221 224 10.1901/jaba.1999.32-221 10396775 PMC1284181

[B19] Campbell MK Elbourne DR Altman DG CONSORT statement: extension to cluster randomised trials BMJ 2004 328 7441 702 708 10.1136/bmj.328.7441.702 15031246 PMC381234

[B20] Fraser M Richman J Galinsky M Day S Intervention research 2009 New York: Oxford University Press

[B21] Baranowski TPC Parcel GS Glanz KRB, Lewis FM How individuals, environments, and health behavoir interact: Social cognitive theory Health behaviour and health education Theory, research, and practice 2002 3 San Francisco: Jossey-Bass

[B22] Wadoodi A Crosby JR Twelve tips for peer-assisted learning: a classic concept revisited Med Teach 2002 24 3 241 244 10.1080/01421590220134060 12098409

[B23] Fixsen D Naoom S Blase K Friedman R Wallace F Implementation research: a synthesis of the literature 2005 Tampa, FA: University of South Florida

[B24] Le Masurier GC Tudor-Locke C Comparison of pedometer and accelerometer accuracy under controlled conditions Med Sci Sports Exerc 2003 35 5 867 871 10.1249/01.MSS.0000064996.63632.10 12750599

[B25] Tudor-Locke C Ainsworth BE Thompson RW Matthews CE Comparison of pedometer and accelerometer measures of free-living physical activity Med Sci Sports Exerc 2002 34 12 2045 2051 10.1097/00005768-200212000-00027 12471314

[B26] Schneider PL Crouter SE Bassett DR Pedometer measures of free-living physical activity: comparison of 13 models Med Sci Sports Exerc 2004 36 2 331 335 10.1249/01.MSS.0000113486.60548.E9 14767259

[B27] Temple VA Stanish HI Pedometer-measured physical activity of adults with intellectual disability: predicting weekly step counts Am J Intellect Dev Disabil 2009 114 1 15 22 19143459 10.1352/2009.114:15-22

[B28] Camelon KM Hadell K Jamsen PT Ketonen KJ Kohtamaki HM Makimatilla S Tormala ML Valve RH The Plate Model: a visual method of teaching meal planning. DAIS Project Group. Diabetes Atherosclerosis Intervention Study J Am Diet Assoc 1998 98 10 1155 1158 10.1016/S0002-8223(98)00267-3 9787722

[B29] Kline P Handbook of psychological testing 2000 London: Routledge

[B30] Durlak JA DuPre EP Implementation matters: a review of research on the influence of implementation on program outcomes and the factors affecting implementation Am J Community Psychol 2008 41 3-4 327 350 10.1007/s10464-008-9165-0 18322790

[B31] Peterson JJ Janz KF Lowe JB Physical activity among adults with intellectual disabilities living in community settings Prev Med 2008 47 1 101 106 10.1016/j.ypmed.2008.01.007 18308385

[B32] Campbell MK Thomson S Ramsay CR MacLennan GS Grimshaw JM Sample size calculator for cluster randomized trials Comput Biol Med 2004 34 2 113 125 10.1016/S0010-4825(03)00039-8 14972631

[B33] Bowling A Research methods in health 2002 2 New York: Open University Press

[B34] Patton M Qualitative research and evaluation methods. (3rd edn.) Thousand Oaks: SAGE; 2002 2002 3 London: Sage Publications, Inc

[B35] Mays N Pope C Qualitative research: Observational methods in health care settings BMJ 1995 311 6998 182 184 7613435 10.1136/bmj.311.6998.182 PMC2550229

[B36] Finlay WM Lyons E Methodological issues in interviewing and using self-report questionnaires with people with mental retardation Psychol Assess 2001 13 3 319 335 10.1037/1040-3590.13.3.319 11556269

[B37] Kvale S Brinkmann S Den kvalitativa forskningsintervjun [The qualitative research interview] 2009 2 Lund: Studentlitteratur

[B38] Malterud K Kvalitativa metoder i medicinsk forskning [Qualitative methods in medical research] 1998 Lund: Studentlitteratur

[B39] Mays N Pope C Qualitative research in health care 1996 London: BMJ Publishing Group

[B40] Anderson LM Quinn TA Glanz K Ramirez G Kahwati LC Johnson DB Buchanan LR Archer WR Chattopadhyay S Kalra GP The effectiveness of worksite nutrition and physical activity interventions for controlling employee overweight and obesity: a systematic review Am J Prev Med 2009 37 4 340 357 10.1016/j.amepre.2009.07.003 19765507

[B41] Wullink M Widdershoven G van Schrojenstein Lantman-de Valk H Metsemakers J Dinant GJ Autonomy in relation to health among people with intellectual disability: a literature review J Intellect Disabil Res 2009 53 9 816 826 10.1111/j.1365-2788.2009.01196.x 19646099

