# Supplementary material for: Promoting a healthy diet and physical activity in adults with intellectual disabilities living in community residences: Design and evaluation of a cluster-randomized intervention
Source: BMC Public Health. 2010 Dec 13;10:761. doi: 10.1186/1471-2458-10-761 (PMC3020685; doi:10.1186/1471-2458-10-761)
Supplement: Additional file 1 — Work routines for meals, physical activity and health. A questionnaire for administrators and managers of community residences concerning health promotion work routines [file 1471-2458-10-761-S1.DOC]

**Routines for meals, physical activity and health**

Questionnaire for administrators and managers of community residences concerning health promotion work routines

**I/We who respond to this questionnaire have the following functions:**

Manager/Head of unit

Work group leader/Coordinator

Staff

Staff with a specific responsibility for health promotion

**Type of residence Special orientation**

Group home No

Supported living  Yes:……………………………………………………

**Type of management Number of residents**:……………......................................

Public

Private **Number of staff**:………………………………………….

Cooperative/association

Questions about general health promoting work

**H1. Health promotion**

Do you conduct health promotion activities with a focus on residents’ health in the residence?

Yes, health promotion directed at residents it is an important part of a policy/action plan and it informs all our work

Yes, health of residents is mentioned in a policy/action plan and/or the staff has an ambition to work with health promotion

No, health promotion has not been a priority, but we have plans to start working with these issues

No

**H2. Staff competence**

Does the staff have competence to carry out health promotion activities targeting the

residents?

*Basic competence* = health care education from upper secondary school or equivalent

*Specific competence* = basic competence including further education in health care/health promotion

Yes, the whole staff has basic competence and some have specific competence

Yes, most of the staff has basic competence and some have specific competence or are about to develop this competence

No, the present competence is insufficient, but there are plans for competence development

No

**H3. Further training of staff**

Is the staff offered in-job training in the areas of food, physical activity and health, through courses, lectures or theme days?

Yes, every year

Yes, at some occasions

No, but there are plans to start offering further training

No

**H4. Ambassadors for health issues**

There are specific ambassadors among staff for any of the following areas (with focus on the health of residents):

- Health
- Recreation and/or physical activity
- Food and meals

Yes, in at least two of the above areas

Yes, in at least one of the above areas

No, but there are plans to introduce ambassadors for these areas

No

**H5. Expert support**

The staff has access to expert support through some of the following:

- Dietitian or municipal food manager
- Nurse or physician
- Physical therapist or occupational therapist
- Health promotion specialist

Yes, from at least three of the above

Yes, from one or two of the above

No, but there are plans to engage expert support

No

**H6. External collaboration**

There is collaboration concerning food, physical activity and health between the residence and some of the following:

- Daily activity center
- Trustees and/or family members
- Habilitation
- The municipality council for culture and leisure

Yes, with at least three of the above

Yes, with one or two of the above

No, but there are plans to start such a collaboration

No

**H7. Health education for residents**

The residents are offered opportunities to develop skills and practices concerning healthy foods and physical activity through some of the following:

- Active support by staff
- Study circles or courses
- Educational material on an appropriate level

Yes, through at least two of the above

Yes, through at least one of the above

No, but there are plans to start offering this

No

**H8. Health problems related to bodyweight**

Is attention paid to residents’ over- and underweight, including health problems related to this?

Yes, attention is paid by the staff, through regular health checks and specific measures when needed

Yes, attention is paid by the staff, through health checks or specific measures when needed

No, but there are plans to start paying attention to this

No

**H9. Alcohol and tobacco**

Is attention paid to alcohol and tobacco habits and health problems associated with alcohol and tobacco among the residents?

Yes, attention is paid by the staff, through regular health checks and specific measures when needed

Yes, attention is paid by the staff, through health checks or specific measures when needed

No, but there are plans to start paying attention to this

No

**H10. Motivation and support for behaviour change**

Do residents who are interested or in need of a change in lifestyle (e.g. improved eating or physical activity) get help with motivation and behaviour change?

Yes, from the staff together with family members or staff at the daily activity center, in addition to expert support (e.g. dietitian or physical therapist)

Yes, from the staff and possibly from someone close to the resident

No, but there are plans to start offering such support

No

Questions about food and meals

**M1. Guidelines/action plans**

There are policies/guidelines for the nutritional content of meals served at the following levels:

- Municipality
- Residence

Yes, at both levels

Yes, at one level

No, but there are plans to develop policies/guidelines for one or both levels

No

**M2. Individual plans for residents**

Are questions regarding healthy foods and meals included in individual plans?

Yes, always if there is a need or if requested

Yes, if the resident, family member or trustee requests it

No, but there are plans to include these questions if there is a need

No

**M3. Meal structure**

In choosing between common and individual meals for the residents are health aspects and the residents´ quality of life considered?

Yes, both the nutritional content of the food and the social wellbeing of all the residents are considered

Yes, we try to do the best possible, although nutritional content and/or social wellbeing cannot always be met for all

No, but we have plans to better consider nutritional content and social wellbeing when choosing between common and individual meals

No

**M4. Meal environment for common meals**

When common meals are served, are they offered in a calm and pleasant environment?

Yes, a great effort is made to create a pleasant environment during the meal and our work routines support this

Yes, there is an ambition to serve meals in a pleasant environment, but we have no specific routines for doing this

No, but there are plans to improve the meal environment

No

Common meals are not served at all

**M5. Nutritional content of common meals**

Are the residents offered healthy meals when common meals are served?

Yes, all meals are planned on the basis of the Swedish nutrition recommendations or advice from the municipality food manager/dietitian

Yes, our ambition is to serve healthy meals

No, but there are plans to start planning healthy meals

No

Common meals are not served at all

**M6. Individual support**

Does the staff give the residents support to help them make healthy choices when they shop and cook?

Yes, the staff always gives the residents support to help them make healthy choices when shopping and cooking, considering the residents´ right to self-determination

Yes, if the resident asks for support

No, but there are plans to start giving such a support

No

**M7. Vegetables and fruits**

Does the staff work actively to promote intake of vegetables and fruits?

Yes, both by offering vegetables/fruits at meals and by supporting the residents to shop and cook vegetables/fruits, considering the residents´ right to self-determination

Yes, we serve vegetables/fruits and/or encourage shopping and cooking vegetables/fruits

No, but there are plans to start promoting intake of fruit and vegetables

No

**M8. Non-core foods and beverages**

Non-core foods and beverages refer to foods which have a low nutrient content but are high in energy (sugar or fat), e.g. candy, sweetened beverages and snacks.

Does the staff work actively to restrict the intake of non-core foods and beverages and to offer healthy alternatives in between meals?

Yes, both by offering healthy alternatives and by supporting the residents to shop and prepare healthy in-between meals, considering the residents´ right to self-determination

Yes, we offer healthy alternatives and/or encourage shopping and preparing healthy in-between meals when there is time and opportunity

No, but there are plans to start to working with this issue

No

Questions about physical activity

**P1. Guidelines/action plans**

Is health promoting physical activity included in guidelines/action plans on some of the

following levels:

- Municipality
- Residence

Yes, at both levels

Yes, at one of the levels

No, but there are plans to develop policies/guidelines for one or both levels

No

**P2. Individual plans of residents**

Are questions regarding health promoting physical activity included in individual

plans?

Yes, always if there is a need or if requested

Yes, if the resident, family member or trustee requests it

No, but there are plans to include these questions if there is a need

No

**P3. Indoor and outdoor physical environment**

The indoor and outdoor physical environment promotes physical activity in some of the following ways:

- By providing access to activating games or exercise equipment for indoor use
- by proximity to garden or other outdoor environment which stimulates activity
- By providing access to activating games, exercise equipment or gardening tools for outdoor use

Yes, through at least two of the above

Yes, through at least one of the above

No, but there are plans to improve the outdoor and indoor environment

No

**P4. Neighborhood environment**

The neighborhood environment supports the possibilities for physical activity in some of the following ways:

- Green areas which invite to outdoor activities
- Safe and illuminated pavements and cycle lanes
- Facilities for physical activity (e.g. pool, gym)
- Activities arranged especially for the target group

Yes, through at least three of the above

Yes, through one or two of the above

No, but there are plans to try to improve the situation

No

**P5. Everyday physical activity**

Are the residents offered support and/or encouragement to be physically active in everyday life (e.g. walking to the shop, choosing the stairs instead of the elevator, working at home or in the garden)?

Yes, the whole staff works actively to give support and encouragement to everyday physical activity, considering the residents´ right to self-determination

Yes, in different ways the staff gives support and encouragement to activities in everyday life

No, but there are plans to start providing such support

No

**P6. Information about leisure time activities including physical activity**

Do the residents on a regular basis get information about leisure time activities including physical activity which they can attend (e.g. swimming, dancing, bowling)?

Yes, the staff actively searches for activities and presents new alternatives every 6 months

Yes, the staff presents activities which they have received information about

No, but there are plans for improving the information given to residents

No

**P7. Support for leisure time activities including physical activity**

Are the residents offered support and opportunities to individual participation in leisure time activities including physical activity?

Yes, the staff works actively to encourage participation in activities, and to accompany residents, if needed

Yes, the staff gives support to participation if requested and if possible

No, but there are plans to develop such a support

No

**P8. Common activities for residents in community residences**

Are the residents offered the opportunity to participate in common activities, initiated by staff, including physical activity, such as walks and excursions?

Yes, on regular scheduled times every week

Yes, sometimes every month, or sometimes more often and sometimes more seldom

No, but there are plans to start such activities

No

Thank you for your time!
